# Synthetic rescue of Xeroderma Pigmentosum C phenotype via PIK3C3 downregulation

**DOI:** 10.1038/s41419-024-07186-4

**Published:** 2024-11-19

**Authors:** Farah Kobaisi, Eric Sulpice, Ali Nasrallah, Patricia Obeïd, Hussein Fayyad-Kazan, Walid Rachidi, Xavier Gidrol

**Affiliations:** 1 Univ. Grenoble Alpes, CEA, Inserm, IRIG, UA13 BGE, Biomics, Grenoble, France; 2 Laboratory of Cancer Biology and Molecular Immunology, Faculty of Sciences I, Lebanese University, Hadath, Beirut, Lebanon

**Keywords:** Nucleotide excision repair, RNAi

## Abstract

Xeroderma Pigmentosum C is a dermal hereditary disease caused by a mutation in the DNA damage recognition protein XPC that belongs to the Nucleotide excision repair pathway. XPC patients display heightened sensitivity to light and an inability to mend DNA damage caused by UV radiation, resulting in the accumulation of lesions that can transform into mutations and eventually lead to cancer. To address this issue, we conducted a screening of siRNAs targeting human kinases, given their involvement in various DNA repair pathways, aiming to restore normal cellular behavior. We introduced this siRNA library into both normal and XPC patient-derived fibroblasts, followed by UVB exposure to induce DNA damage. We assessed the reversal of the XPC phenotype by measuring reduced photosensitivity and enhanced DNA repair. Among the 1292 kinase-targeting siRNAs screened, twenty-eight showed significant improvement in cellular survival compared to cells transfected with non-targeting siRNA after UV exposure in XPC cells. From these candidates, PIK3C3 and LATS1 were identified as particularly effective, promoting over 20% repair of 6-4 photoproduct (6-4PP) DNA lesions. Specifically targeting the autophagy-related protein PIK3C3 alone demonstrated remarkable photoprotective effects in XPC-affected cells, which were validated in primary XPC patient fibroblasts and CRISPR-Cas9 engineered XPC knockout keratinocytes. PIK3C3 knock down in XP-C cells ameliorated in UVB dose response analysis, decreased apoptosis with no effect on proliferation. More importantly, PIK3C3 knock down was found to induce an increase in UVRAG expression, a previously reported cDNA conveying lower photosensitivity in XP-C cells. Thus, attempts to improve the XPC photosensitive and deficient repair phenotype using PIK3C3 inhibitors could pave a way for new therapeutic approaches delaying or preventing tumor initiation.

## Introduction

UV radiation leads to significant damage to DNA. UVB rays (with wavelengths between 280 and 320 nm) directly cause the formation of pyrimidine dimers in DNA, such as Cyclobutane Pyrimidine Dimers (CPD), 6-4 pyrimidine-pyrimidone photoproducts (6-4PP), and Dewar isomers, which can result in double strand breaks during replication [1]. A notable mutation induced by UVB is the transition of cytosine (C) to thymine (T) [2]. UV-A radiation indirectly harms DNA through the generation of reactive oxygen species (ROS) [3]. DNA repair primarily relies on nucleotide excision repair (NER), which includes Global Genome Repair (GGR) and Transcription Coupled Repair (TCR). GGR starts with the recognition of lesions by protein complexes like XPC-Rad23B-Centrin2 and XPE-DDB1, while TCR involves proteins such as CSA and CSB for recognition. Repair processes involve enzymes like helicases, nucleases, and DNA polymerase [4–6]. Deficiencies in NER can lead to disorders such as Xeroderma Pigmentosum (XP), characterised by various symptoms. For instance, the XP-C group exhibits mutations in the GGR damage recognition protein, resulting in DNA lesions, photosensitivity, and an elevated risk of early skin tumors [7]. Certain mutations, like suppressor mutations, can alleviate the effects of primary mutations [8]. For example, in Fanconi Anemia, the suppression of the RecQ-like DNA helicase BLM complex serves as a mitigating factor [9]. Similarly, inhibiting Adenine DNA glycosylase MUTYH shows promise as a treatment strategy in Xeroderma Pigmentosum A [10]. This study aims to identify suppressor mutations in XP-C by screening patient cells with a kinase-targeting siRNA library under UVB exposure, with the intention of discovering novel therapeutic interventions.

## Results

### Characterization of WT and XP-C patient derived fibroblast cell lines

**Fig. 1:**
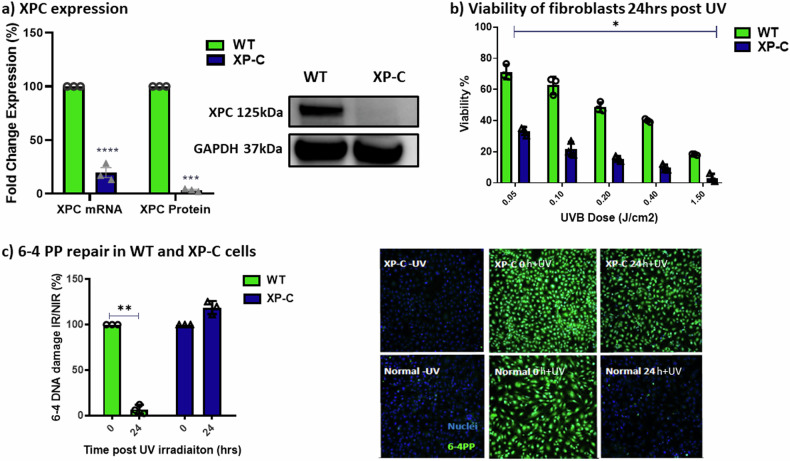
Characterization of the XP-C and WT cells. The expression of XPC in both cell lines was examined along with the cells’ photosensitivity and repair capacity. **a** XPC expression. XPC mRNA is five-fold lower in XP-C cells. At the protein level, western blot analysis revealed the total absence of XPC protein expression in XP-C cells, unlike the WT cells. **b** Viability of fibroblasts 24 h post UVB. XP-C cells manifest significantly increased photosensitivity compared to WT cells (**c**) 6-4PP repair in Normal and XP-C cells. 6-4 PP quantification was conducted on irradiated or non-irradiated WT and XP-C cells. XP-C cells show elevated levels of DNA damage 24 h post UV while normal cells show significant repair at the same time point. Single cell analysis was carried out via the quantification of nuclear DNA damage in several individual cells per condition. Hoechst staining was utilised for the identification of the nuclei that will be set as the region of interest (ROI) for the quantification of DNA damage. DNA damage at time zero was set as 100% while that of non-irradiated cells was set as 0% damage. *N* = 3 Mean ± S, Student T test, *p-value < 0.05, **p-value < 0.01, ***p-value < 0.001.

XPC mRNA and protein expression levels were assessed in both wild-type (WT) and XP-C immortalised patient fibroblasts. In XP-C cells, XPC mRNA was found to be decreased by five-fold compared to WT cells, and there was a complete absence of XPC protein (Fig. 1a, Supplementary File 1). Additionally, XP-C cells exhibited significantly lower viability in response to increasing doses of UVB radiation compared to WT cells, as observed in the UVB dose-response analysis conducted 24 h post-irradiation (Fig. 1b). Furthermore, analysis of UVB-induced 6-4 photoproducts (6-4PP) by immunocytochemistry (ICC) showed that XP-C cells were unable to repair the damage, unlike WT cells which displayed complete repair 24 h post-UV exposure (Fig. 1c).

### Identification of LATS1 and PIK3C3 whose knock down partially reverses XP-C photosensitivity and DNA damage accumulation

**Fig. 2:**
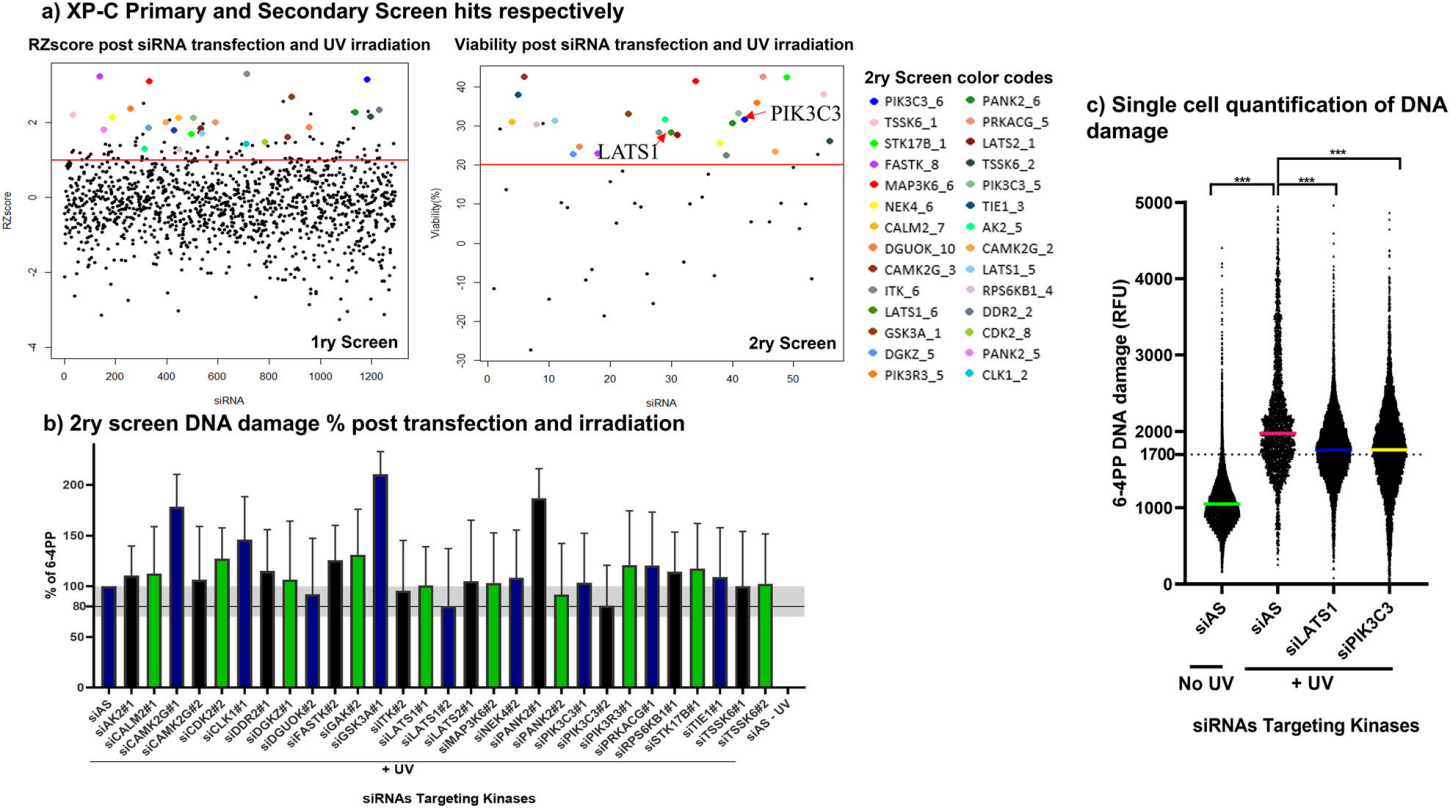
Identification of LATS1 and PIK3C3 whose knockdown partially reverses XP-C photosensitivity and damage accumulation. The screening of kinase siRNA library on XP-C and WT cells allowed the identification of kinases, whose knock down can enhance the viability of XP-C cells. **a** Primary XPC screen hits were chosen on the basis of RZscore ≥ 1.8. The kinases chosen were subsequently validated in secondary screen with two siRNAs per kinase and the hits were those showing viability > 20%. The color codes of only the secondary screen hits are displayed in the legend. **b** Percent of DNA damage post siRNA transfection. Kinases whose knockdown enabled a 20% decrease in DNA damage were selected, among them, LATS1 and PIK3C3. **c** Single cell quantification of DNA damage. *N* = 3 Mean ± SD, Student T test *** p-value < 0.001.

To identify kinases whose downregulation could alleviate the photosensitivity of XP-C cells, we conducted an RNA interference (RNAi) assay using a siRNA library targeting all human kinases, with two siRNAs designed for each kinase. A scrambled non-targeting siRNA (siAllstar, siAS) served as a negative control, while a mixture of siRNAs inducing cell death acted as the positive control. Both XPC-mutated and wild-type (WT) fibroblasts were transfected with the siRNA libraries for 48 h before being exposed to UVB irradiation at 0.03 J/cm2. Cell viability was assessed 24 h post-UVB irradiation. Twenty-eight kinases were identified in the primary screen based on their knockdown reducing cell death compared to the scrambled siRNA, exhibiting a robust Z score greater than 1.8. These hits underwent validation through secondary screening, with two siRNAs tested per kinase, and hits were defined as those showing viability of at least 20% (Fig. 2a).

Subsequently, DNA damage analysis using 6-4 photoproduct (6-4PP) antibody staining was performed on XP-C cells transfected with the identified hits, using two siRNAs per kinase. The positive control demonstrated maximal DNA damage in siAS-transfected irradiated cells, while the negative control exhibited minimal damage in non-irradiated siAS-transfected cells. siRNAs targeting the genes LATS1 and PIK3C3 enabled a 20% repair of DNA damage compared to the control siAS-transfected irradiated cells (Fig. 2b). This DNA damage repair was particularly significant for these two kinases, as demonstrated by the quantification of 6-4PP at the single-cell level (Fig. 2c). The on-target knockdown efficiency of the latter siRNAs was validated at both mRNA and protein levels, accompanied by in silico analysis of off-target effects (Supplementary Fig. 1). Thus, these two siRNAs were chosen because, in addition to increasing viability, they also facilitated DNA damage repair, unlike other siRNAs that enhanced viability but failed to mediate DNA damage repair.

### PIK3C3 knock down is exclusively photo-protective to XP-C cells and not WT

**Fig. 3:**
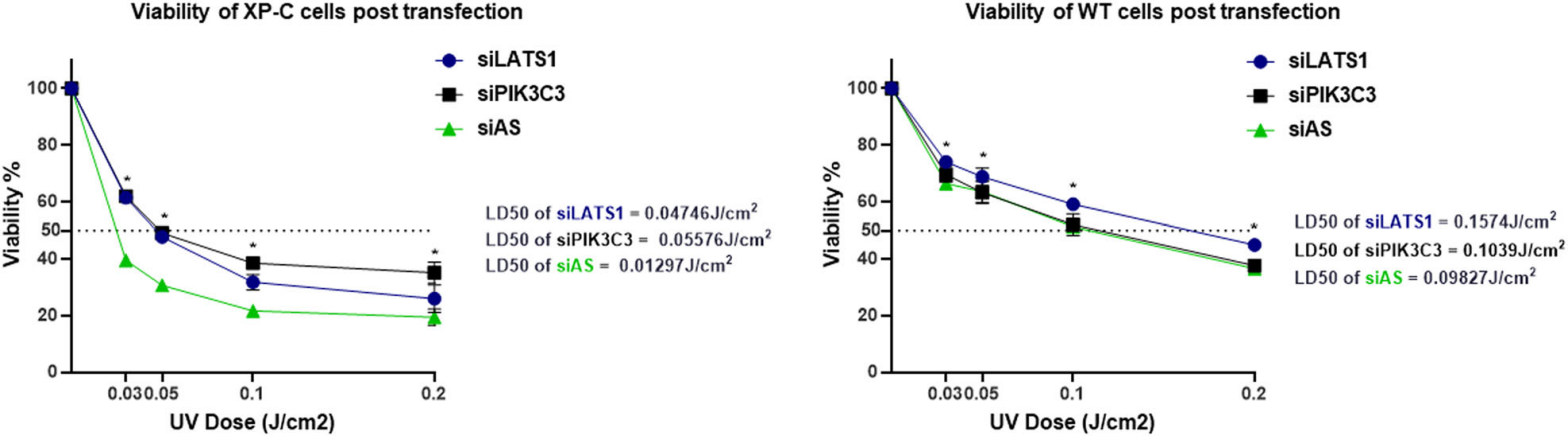
Knockdown of PIK3C3 has exclusive protective effect in XP-C cells while LATS1 can protect both WT and XP-C cells from UVB induced cell death. XP-C and WT cells were transfected with either siLATS1, siPIK3C3 or siAS, before being subjected to increased UVB doses. Both siRNAs demonstrated UV-protection in XP-C cells, with siPIK3C3 exhibiting a notably higher and statistically significant enhancement in XP-C viability compared to siAS. WT cells UVB-protection was only evident in cells with LATS1 knockdown while siPIK3C3 transfection had no effect. N = 3 Mean ± SD, Student T test *p-value < 0.05.

To further investigate the impact of PIK3C3 and LATS1 knockdown on enhancing cell viability, both XP-C and WT cells were transfected with either siAS, siPIK3C3, or siLATS1, then exposed to escalating doses of UVB radiation. After 24 h, viability was assessed using the Presto Blue assay and normalised to that of non-irradiated cells, set at 100%. In XP-C cells, transfection with siPIK3C3 significantly conferred protection, with an LD50 of 0.056 J/cm^2^ against increasing UVB doses, compared to control siAS-transfected cells which displayed an LD50 of 0.013 J/cm^2^. However, this effect was not observed in WT cells, where only siLATS1 transfection enhanced post-UVB viability (Fig. 3).

### Knockdown phenotypic outcome at the level of apoptosis and proliferation

**Fig. 4:**
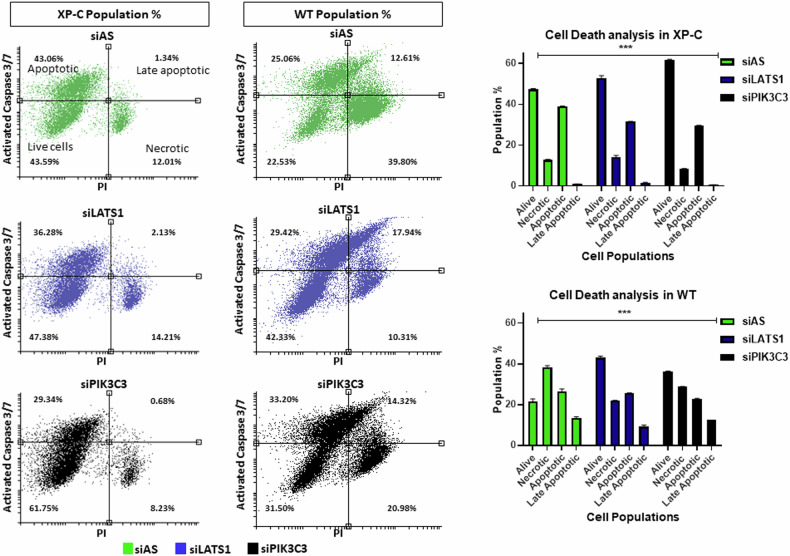
Physiological consequence of LATS1 and PIK3C3 knock down on the level of apoptosis. Analysis of apoptosis levels in XP-C and WT cells post transfection. Both knock downs efficiently increased live cell populations in XP-C cells, with siPIK3C3 mediating a higher increase. For WT cells, LATS1 knock down mediated a higher increase in live cell population compared to siPIK3C3 or siAS. *N* = 3 Mean ± SD, Repeated measure *** *p*-value < 0.001.

To explore the cellular mechanisms influenced by the downregulation of the selected kinases LATS1 and PIK3C3, we conducted further analysis focusing on cell death and proliferation. XP-C or WT cells were transfected with either siAS, siLATS1, or siPIK3C3 for 48 h, followed by irradiation at doses of 0.1 J/cm^2^ for WT cells and 0.02 J/cm^2^ for XP-C cells, considering their higher photosensitivity. After twenty hours post-UVB irradiation, the cells were stained and analyzed. Both siLATS1 and siPIK3C3 transfections led to a significant increase in the population of live XP-C cells (identified by low caspase 3/7 activity and propidium iodide negativity) compared to cells transfected with control siAS. Gating controls are provided in Supplementary Fig. 4. The photoprotection conferred by PIK3C3 knockdown in XP-C cells was observed to be more pronounced than that achieved by LATS1 knockdown. Conversely, in WT cells, knockdown of LATS1 resulted in a greater increase in the live cell population compared to the relatively lower increase induced by PIK3C3 knockdown (Fig. 4). This indicates that PIK3C3 knockdown in XP-C cells and LATS1 knockdown in WT cells decrease the level of apoptosis in these cells following UVB irradiation. Furthermore, quantification of proliferative capacity two hours post-UV exposure and EdU incorporation in transfected XP-C and WT cells revealed no significant variation for both kinases downregulation (Supplementary Fig. 2).

### SiPIK3C3 and siLATS1 also exert protective effects on CRISPR-Cas9 engineered XPC knock out immortalised keratinocytes

**Fig. 5:**
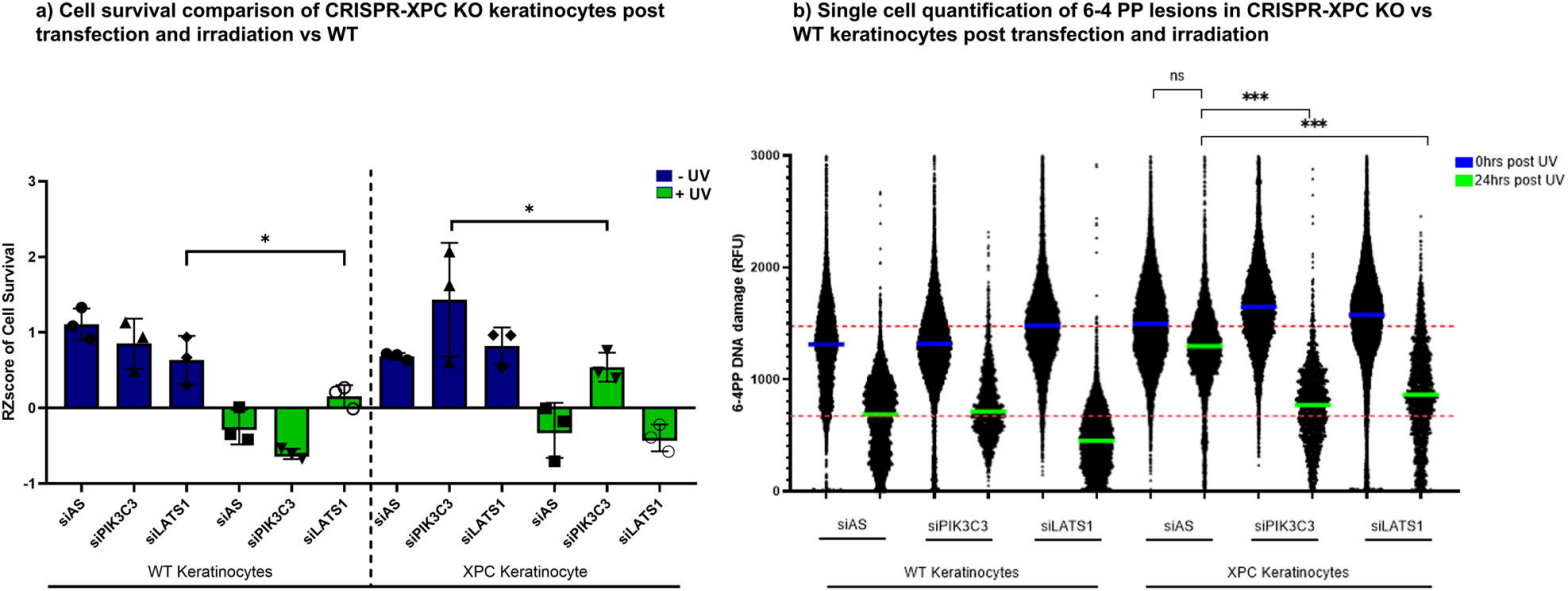
PIK3C3 also rescue in XPC-KO CRISPR generated keratinocyte cells increasing cells viability and decreasing DNA damage. XP-C-KO and WT keratinocytes cells were transfected with either siLATS1, siPIK3C3 or siAS and then subjected to increased UVB doses. **a** PIK3C3 siRNAs showed UV-protection in XP-C cells viability in comparison to siAS. WT cells UVB-protection was evident in cells with LATS1 knockdown, while siPIK3C3 transfection had no effect. **b** The knock down of both kinases enabled a significant decrease in DNA damage in XP-C keratinocytes when compared with siAS cells. *N* = 3 Mean ± SD, Student T test *p-value < 0.05, ***p-value < 0.001.

In the pathology of XPC disease, keratinocytes play a crucial role as they are the outermost cells of the skin exposed to UV irradiation and are implicated in the development of most skin carcinomas. Due to the limited availability of XPC keratinocytes, we generated hTERT immortalised keratinocyte cell lines with a knockout of the XPC gene using CRISPR-Cas9 technology. Sequencing details are described in supplementary Fig. 5. Both wild-type (WT) and XPC-knockout (XPC-KO) keratinocytes were transfected with either siAS, siPIK3C3, or siLATS1 and subsequently irradiated. After irradiation, we assessed cell viability using the Presto Blue assay and quantified DNA damage by staining the cells with 6-4 photoproduct (6-4PP) antibody. WT keratinocytes with knockdown of LATS1 mRNA exhibited a significant increase in cell survival, whereas XPC-KO cells with knockdown of PIK3C3 showed a significant increase in viability (Fig. 5a). Additionally, the knockdown of both PIK3C3 and LATS1 in XPC-KO cells resulted in a significant decrease in the levels of 6-4PP compared to XPC-KO cells transfected with siAS (Fig. 5b). However, only one clone of XPC-KO keratinocytes was tested so further testing of the effect of knock out on other clones will be envisioned.

### Effect of LATS1 downregulation on YAP translocation

**Fig. 6:**
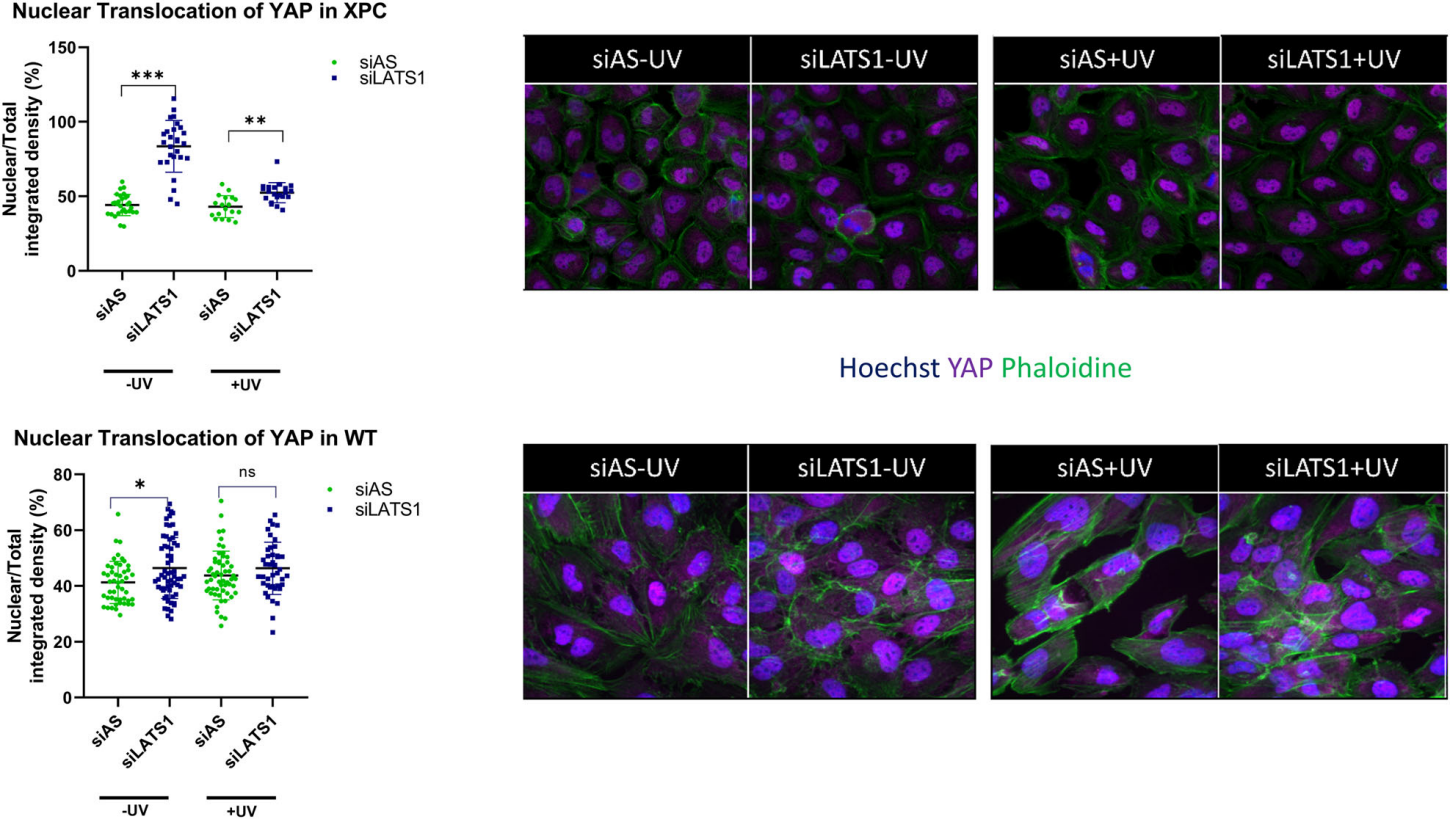
LATS1 knockdown enhances YAP translocation in XP-C cells. XP-C cells manifested increased translocation of YAP to nuclei prior and post UV upon LATS1 knockdown. WT cells also showed YAP translocation at the basal no UV level that was less upon irradiation. *N* = 3 Mean ± SD, Student T test, * *p*-value < 0.05, ** *p*-value < 0.01, *** *p*-value < 0.001.

LATS1 functions as a negative regulator of Yes-associated protein 1 (YAP), inhibiting its translocation into the nuclei and thereby suppressing transcription [11]. We quantified YAP translocation following LATS1 downregulation. XP-C and WT cells were seeded in 96-well plates and transfected with either siAS or siLATS1 for 48 h. One hour post-irradiation, the cells were fixed and incubated with an antibody against YAP. Phalloidin staining was used to outline the cell circumference, while Hoechst staining facilitated the delineation of the nuclear region. Upon LATS1 knockdown, XP-C cells exhibited significant translocation of YAP into the nuclei at baseline, which decreased upon irradiation but remained significantly higher compared to siAS-transfected irradiated XP-C cells. Conversely, WT cells showed a slight increase in YAP translocation to the nuclei at both baseline and post-irradiation states, but this increase was not statistically significant (Fig. 6). The translocation of YAP into the nuclei elucidates the observed increase in viability (Fig. 3) following siRNA-mediated depletion of LATS1 in both cell lines.

### PIK3C3 knockdown mediated synthetic rescue specifically in XP-C cells

**Fig. 7:**
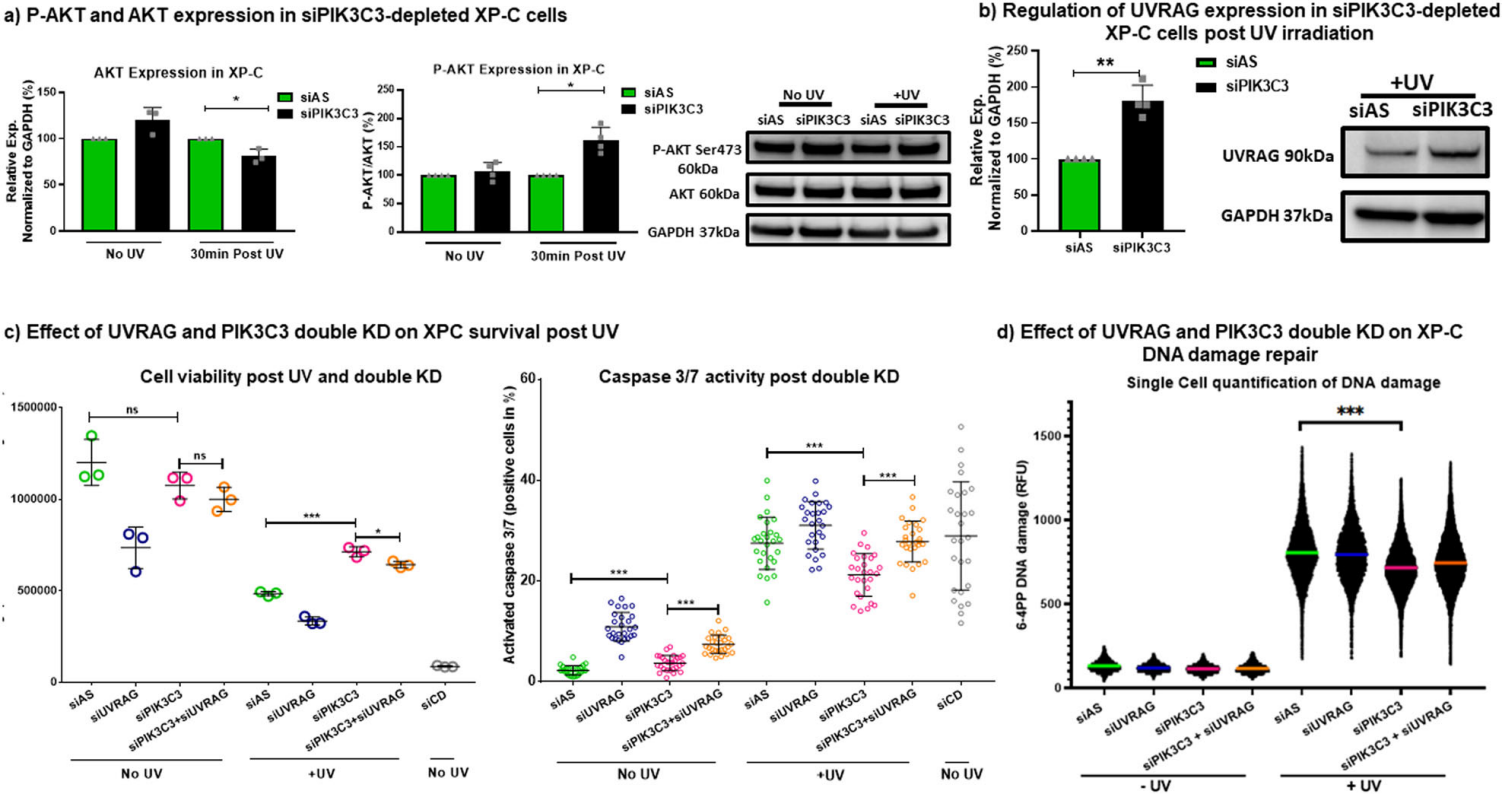
Synthetic rescue of XP-C cells by PIK3C3 knockdown is mediated by decreasing AKT levels while enhancing the levels of P-AKT Ser473 and UVRAG*.* **a** P-AKT and AKT expression in XP-C siPIK3C3 cells. An increase of P-AKT Ser473 was detected in XP-C cells post irradiation accompanied by a decrease in AKT levels. **b** Regulation of UVRAG expression in XP-C. The cells manifest a significant increase in UVRAG expression upon PIK3C3 deregulation. **c** The combined loss of both UVRAG and PIK3C3 results in a reduction in cell viability, as indicated by Presto Blue assay. Additionally, analysis of cell death through caspase 3/7 activity staining reveals an increase in apoptotic cell population when PIK3C3 knockdown is combined with UVRAG loss. **d** Effect of UVRAG and PIK3C3 double loss on XP-C DNA damage repair. An increase in the levels of DNA damage in the double loss condition signifying the loss of the PIK3C3 mediated rescue upon UVRAG knockdown. *N* = 3 Mean ± SD, Student T test, *p-value < 0.05, **p-value < 0.01, ***p-value < 0.001.

UVB irradiation has been documented to induce the phosphorylation of AKT Ser473, contributing to skin survival through the inhibition of apoptosis [12]. To investigate this, we assessed the expression levels of both AKT and phosphorylated AKT Ser473 (P-AKT Ser473). In XP-C cells, basal AKT expression showed a non-significant increase in siPIK3C3-transfected cells. However, post-irradiation, AKT expression decreased in siPIK3C3-transfected XP-C cells, accompanied by a significant rise in AKT Ser473 phosphorylation compared to siAS controls (Fig. 7a, Supplementary File 1). Conversely, WT cells with PIK3C3 downregulation exhibited no significant changes in AKT or P-AKT Ser473 expression levels at baseline or post-irradiation (Supplementary Fig. 3a). This lack of differential expression and phosphorylation supports the notion that siPIK3C3 transfection does not affect apoptosis in WT cells as it does in XP-C cells.

PIK3C3 plays a pivotal role in autophagy by forming complexes with Beclin [13] and UVRAG, with the latter being associated with DNA damage repair [14]. Thus, we investigated UVRAG levels following PIK3C3 knockdown and irradiation. XP-C and WT cells transfected with siPIK3C3 or siAS were irradiated, and protein extracts were analyzed via blotting. UVRAG was significantly upregulated in XP-C cells with PIK3C3 knockdown, while WT cells did not exhibit any change in UVRAG expression (Fig. 7b, Supplementary File 1, Supplementary Fig. 3b). To validate the role of UVRAG in mediating XP-C rescue downstream of PIK3C3 knockdown, we conducted double loss-of-function experiments targeting both PIK3C3 and UVRAG mRNA with siRNAs. XP-C cells were transfected with siAS, siPIK3C3, siUVRAG, or siPIK3C3+siUVRAG for 2 days, then irradiated and further incubated for 24 hours. The effect of knockdown on cell survival was quantified using Presto Blue or CellEvent caspase 3/7 activity staining. A slight decrease in the ability of PIK3C3 knockdown to mediate XP-C cell survival was observed upon combined knockdown of UVRAG post-UV exposure. This effect was more pronounced when measuring caspase 3/7 activity, indicating an increase in apoptotic cells. The percentage of apoptotic cells versus the total cell count stained with DAPI was calculated for all conditions. The lowest percentage of apoptotic cells was observed with PIK3C3 knockdown alone, further validating the rescue effect of PIK3C3 knockdown in XP-C cells. However, upon double knockdown of PIK3C3 and UVRAG, the percentage of apoptotic cells increased to levels similar to siAS-transfected cells, masking the rescue effect mediated by PIK3C3 knockdown alone (Fig. 7c). Similarly, the same protocol was followed to assess DNA damage levels using 6-4 PP antibody staining. PIK3C3 knockdown decreased DNA damage levels in XP-C cells compared to siAS. However, when UVRAG was knocked down together with PIK3C3, DNA damage levels tended to increase (Fig. 7d). Thus, this double loss experiment confirmed the involvement of UVRAG in the rescue effect mediated by PIK3C3 knockdown in XP-C cells.

## Discussion

This study aimed to mimic suppressive alterations and identified two kinases whose knockdown can partially reverse the phenotype of XP-C cells, leading to increased resistance to UVB radiation and decreased accumulation of DNA damage following irradiation. These kinases are LATS1, a key player in the Hippo pathway, and PIK3C3, primarily involved in autophagy. The effect of LATS1 knockdown was observed in both WT and XP-C cells, whereas PIK3C3 downregulation conferred photoprotection exclusively to XP-C cells.

Increased cell viability alone, without repair, can potentially lead to the accumulation of cells with DNA lesions and ultimately contribute to carcinogenesis [15], thus justifying the use of these dual readouts. Phenotypic reversal, particularly in terms of increased viability post-UV exposure, may arise from modulation of apoptosis or cell proliferation. In this study, it appears to predominantly involve the reduction of apoptosis for both WT and XP-C cells with LATS1 downregulation, and only in XP-C cells with PIK3C3 downregulation. However, one limitation of our study is that one siRNA for either LATS1 or PIK3C3 was utilized downstream the screening to further characterize the phenotypic reversal.

AKT is a crucial effector of the PI3K/AKT/mTOR pathway, regulating various functions including anti-apoptosis, proliferation, and DNA repair. Its activation involves phosphorylation of Thr308 by PDK1 [16] and Ser473 by mTORC2 [17] and DNA-PK [18]. Post-UVB irradiation, upregulation of P-AKT Ser473 was observed in XP-C cells transfected with siPIK3C3. Tu et al. demonstrated that AKT Ser473 phosphorylation requires the association of DNA-PKcs-mTORC2 upon UVB irradiation and is attenuated by the depletion of either, reducing UVB-induced cell death and apoptosis [12]. This mechanism could potentially explain the anti-apoptotic effect mediated by different transfections via the upregulation of AKT Ser473 phosphorylation. Another possible mechanism involves P300 histone acetyltransferase activated by AKT, facilitating chromatin remodeling and recruitment of repair complexes, thereby decreasing the need for cell death commitment [19].

The actual link between LATS1 or PIK3C3 downregulation and increased viability accompanied by reduced damage requires further investigation. Regarding LATS1, nuclear translocation is a straightforward downstream mechanism to analyze. XP-C cells exhibited increased nuclear translocation upon siLATS1 transfection, albeit to a lesser extent in WT cells. However, this translocation was not associated with changes in the proliferative capacity of XP-C cells, suggesting the involvement of another downstream effector favored by YAP nuclear translocation.

PIK3C3 plays a pivotal role in autophagy regulation, where it forms complexes with various partners including Beclin and UVRAG [13]. We aimed to assess the impact of PIK3C3 downregulation on downstream UVRAG expression. XP-C cells exhibited upregulation of UVRAG expression following PIK3C3 downregulation, a phenomenon not observed in WT cells. This suggests that the increased UVRAG expression in siPIK3C3-transfected XP-C cells might be mediating a photoprotective effect independent of its role in autophagy. Intriguingly, UVRAG was initially identified as a cDNA capable of partially complementing UV sensitivity in Xeroderma Pigmentosum C cells [20, 21]. Further investigation revealed its involvement in GG-NER, where it accumulates at lesions and associates with DDB1 to facilitate the assembly and activity of the CRL4DDB2 complex. This complex ubiquitinates downstream NER factors and histones, facilitating the recruitment of downstream effectors and destabilisation of damage-containing nucleosomes [22]. Additionally, UVRAG is implicated in centrosome stability and DNA-PK regulation [14]. The upregulation of UVRAG expression in XP-C cells following PIK3C3 downregulation might result from decreased AKT expression [23]. The concurrent loss of UVRAG with PIK3C3 knockdown renders the viability and DNA damage levels of XP-C cells similar to those of siAS, indicating a partial loss of the rescue effect mediated by PIK3C3 knockdown upon UVRAG loss. Further investigation into UVRAG accumulation at damage sites is warranted to elucidate its autophagy-independent role in the XPC-deficient model of GG-NER.

Although more research is needed, the synthetic rescue of XP-C cells by siPIK3C3 suggests promising therapeutic avenues. Noman et al. demonstrated that genetic suppression of PIK3C3 or treatment with selective inhibitors of its kinase activity in melanoma and colorectal tumor cells could reprogram tumors from cold to warm inflamed tumors infiltrated by the immune system [24]. This study provides proof of concept for innovative clinical trials targeting cold tumors unresponsive to immune checkpoint blockade by combining PIK3C3 inhibitors with anti-PDCD1/PD-1 and anti-CD274/PD-L1. Moreover, it would be intriguing to investigate whether autophagy activation in XP-C cells, alongside DNA repair deficiency, contributes to the significantly increased risk of melanoma in XP-C patients. Additionally, exploring the potential of PIK3C3 inhibitors alone or in combination with immune checkpoint inhibitors to reduce this risk warrants further investigation.

## Materials and methods

### Cell lines

Wild-type (AG10076) and XP-C (GM15983) immortalised patient-derived fibroblasts were obtained from the Coriell Biorepository (Coriell Institute, New Jersey, USA). These cells were cultured in DMEM high glucose, GlutaMAX media (Gibco, Massachusetts, USA), supplemented with 10% FBS and 1% penicillin/streptomycin, and maintained at 37 °C in a 5% CO_2_ incubator.

To generate XPC knockout (XPC-KO) cell lines, hTERT immortalised keratinocytes were utilised. Briefly, the keratinocytes were nucleofected with cas9 protein and XPC-targeting gRNA (ThermoFisher Scientific). Following electroporation, clonal expansion of the cells was performed to isolate a single clone of keratinocytes lacking XPC expression at both the RNA and protein levels [25].

### siRNA Kinome Targeting Library

A siRNA library targeting all human kinases (Qiagen, Hilden, Germany) was employed in this study. This library comprises two siRNAs targeting each of the 646 human kinases, totaling 1292 siRNAs. Screening was conducted in 96-well plates, with one siRNA transfected per well at a final concentration of 5 nM. Transfection was carried out using Lipofectamine RNAiMax (ThermoFisher Scientific, Massachusetts, USA).

The sequences of hit siRNAs are CGCGATCTAGTATATGTTTAA for LATS1 and TCGGTTGGTGCATCTAATGAA for PIK3C3.

### UVB dose–response

We investigated the photosensitivity of XP-C cells relative to WT cells in response to increasing UVB doses. Cells were seeded in 96-well plates until reaching 80% confluency, followed by PBS washing and exposure to escalating UVB doses. After 24 h of UVB irradiation, cell viability was assessed using PrestoBlue (ThermoFisher Scientific) following the manufacturer’s instructions. Normalisation of the data involved calculating the percentage of viability at each dose relative to the viability of control non-irradiated cells (dose zero), which was set as 100% viability. The experiment was carried out for *N* = 3 with three technical replicates.

### 6–4PP DNA damage staining and quantification

DNA damage quantification was performed by immunostaining the cells with an antibody against 6-4 PP (Cosmo Bio, California, USA), followed by a secondary antibody, mouse AF488 (Invitrogen, California, USA). Cells were seeded until reaching confluency and then exposed to UVB irradiation at 0.03 J/cm^2^. Twenty hours after irradiation, the cells were stained according to a previously established protocol [26]. Image acquisition was conducted at 10× magnification, followed by quantification using Cell-Insight NXT. For each tested condition ≥5000 cells were analyzed.

### EdU incorporation-cell proliferation assay

To assess the impact of transfection on cell proliferation, both cell lines were cultured in 6-well plates and transfected with either siAS, siLATS1, or siPIK3C3. After 48 h of transfection, the cells were exposed to irradiation at 0.02 J/cm^2^ and then allowed to incubate for appropriate durations in the presence of EdU. Subsequently, the cells were harvested, stained according to the manufacturer’s protocol, and subjected to analysis by flow cytometry using a BD LSRII flow cytometer (BD Biosciences). Post-analysis was conducted using Flowing Software [27] (Turku Bioimaging, Finland). The experiment was carried out for *N* = 3 with three technical replicates.

### Cell death quantification

Forty-eight hours after transfection, cells in 6-well plates were irradiated and subsequently incubated with both CellEvent (ThermoFisher Scientific, Massachusetts, USA), a caspase 3/7 activity probe, and propidium iodide (PI). After incubation, the cells were collected and analyzed by flow cytometry using a BD LSRII flow cytometer (BD Biosciences) twenty-four hours post-irradiation at 0.02 J/cm^2^ and 0.1 J/cm^2^ for XP-C and WT cells, respectively. Additional analysis was conducted using Flowing Software [27] (Turku Bioimaging). The experiment was carried out for *N* = 3 with three technical replicates.

### qRT-PCR

Total RNA extraction was conducted using the RNeasy Plus Mini Kit (Qiagen), followed by quantification using the Nanodrop 1000. Subsequently, 1 µg of RNA was reverse transcribed to cDNA utilising the SuperScript IV VILO Master Mix (Invitrogen). Next, 5 µL of cDNA (5 ng/µL) was utilised in qPCR reactions with gene-specific primers targeting XPC, PIK3C3, LATS1, FOXN1, and GAPDH (Qiagen). The qPCR was performed using Platinum SYBR Green qPCR SuperMix-UDG (Invitrogen). The primer sequences used were as follows: XPC (CCATGAGGACACACACAAGG, TCCAATGAACCACTTCACCA), UVRAG (CTT GGG TCA GCA GAT TCA TGC, CGT AAG AAT TGC GAA CAC AG), LATS1 (#QT00096943), FOXN3 (#QT00049525), PIK3C3 (#QT00074004). Samples were run in triplicates through the BioRad CFX96 Real-time System (C1000 Touch Thermal Cycler). Relative expression levels were calculated using the 2-ΔΔCT method as reported by Livak et al. [28].

### Protein extraction and Immunoblotting

Proteins were extracted using RIPA buffer (Sigma Aldrich), supplemented with protease and phosphatase inhibitors. Western blotting was conducted according to previously established protocols [29]. Images were captured using the Biorad Molecular Imager® Chemi DocTM XRS system and analyzed using Image LabTM software. For phosphorylation experiments, the membranes were stripped using ReBlot Plus Mild Antibody Stripping Solution (Merck, Darmstadt, Germany), washed, blocked with 5% BSA, and subsequently re-incubated with primary antibodies following the aforementioned protocol. The experiment was carried out for *N* = 3. The used primary antibodies with their references and dilutions are detailed in supplementary table 1.

### Immunofluorescence and associated microscopy

To investigate the translocation of YAP, both WT and XP-C cells were seeded in 96-well microplates. These cells were transfected with either siAS or siLATS1, followed by irradiation at a dose of 0.02 J/cm^2^. One hour post-irradiation, the cells were fixed and subsequently stained with an anti-YAP antibody (Santa Cruz, Texas, USA). A secondary antibody, mouse-C5 antibody (Jacksons Lab, Maine, USA), was then incubated along with FITC-Phalloidin (ThermoFisher Scientific, Massachusetts, USA). Nuclear DNA was counter-stained with Hoechst (Sigma-Aldrich, Missouri, USA). Cell images were acquired using the Zeiss LSM880 Microscope at 40X magnification. For each condition ≥ 100 cell were quantified.

### Statistical analysis

Screening hit selection and single cell analysis were carried out by R software [30]. GraphPad Prism v.8 was utilised for statistical analysis.

## Supplementary information

Supplementary Table 1

Supplementary Figures

Reproducibility Check list

Supplemental uncropped western

## Data Availability

Data sharing not applicable to this article as no datasets were generated or analyzed during the current study.
