# Supplementary material for: Synthetic rescue of Xeroderma Pigmentosum C phenotype via PIK3C3 downregulation
Source: Cell Death Dis. 2024 Nov 19;15(11):847. doi: 10.1038/s41419-024-07186-4 (PMC11577109; doi:10.1038/s41419-024-07186-4)
Supplement: Supplementary file 2 — Supplementary Figures [file 41419_2024_7186_MOESM2_ESM.pdf]

Supplementary Figures

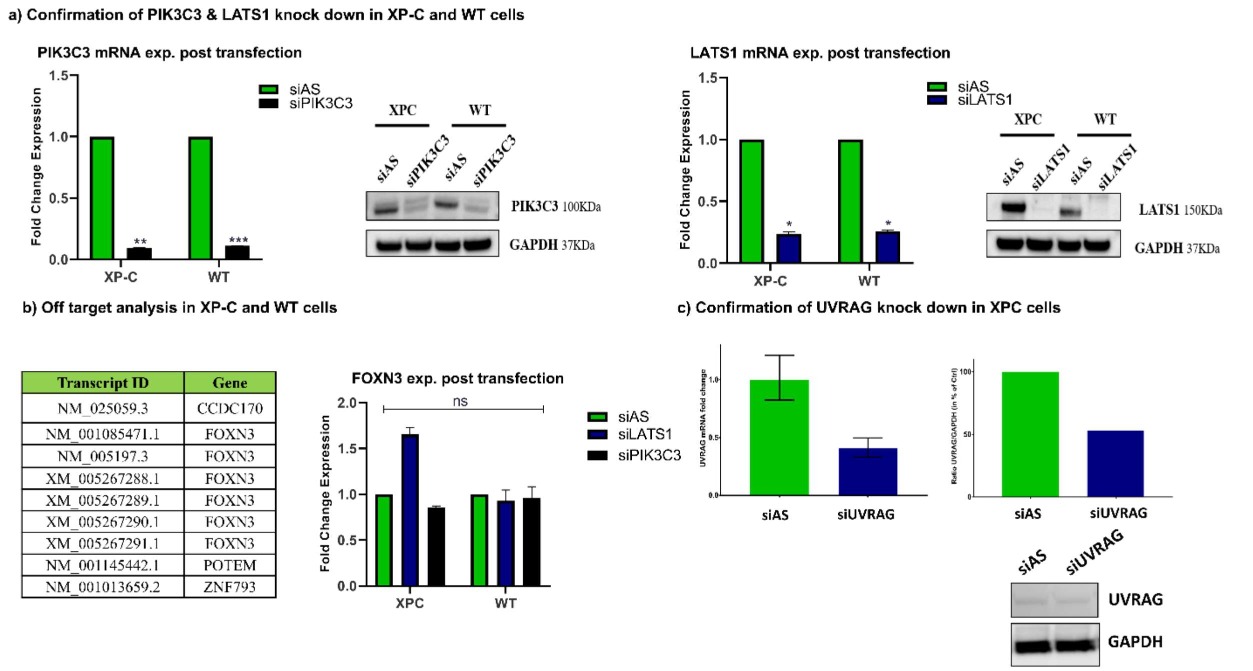

Transcript ID	Gene
NM_025059.3	CCDC170
NM_001085471.1	FOXN3
NM_005197.3	FOXN3
XM_005267288.1	FOXN3
XM_005267289.1	FOXN3
XM_005267290.1	FOXN3
XM_005267291.1	FOXN3
NM_001145442.1	POTEM
NM_001013659.2	ZNF793

Supplementary Figure 1 Validation of on and off targets of LATS1 and PIK3C3 as well as on target analysis of UVRAG siRNA.

The validation of both the on and off target effects of both siLATS1 and siPIK3C3 was carried out. a) Confirmation of target gene knock down in XP-C and WT cells. To ensure that both siPIK3C3 and siLATS1 specifically knockdown the expression of their targets, XP-C and WT cells were transfected with either these siRNAs or siAS. After forty eight hours of incubation both RNA and protein extraction were carried out. Both siPIK3C3 and siLATS1 were able to significantly decrease the expression of PIK3C3 and LATS1 respectively at the mRNA level in both XP-C and WT detected by RT-PCR. This knockdown was validated at the level of protein expression via western blot where transfected cells manifest absence of protein bands at the level of each target protein compared to siAS transfected samples. b) Off-target analysis in XP-C and WT cells. siRNAs can possess miRNA like effects on downregulating genes other than their targets based on incomplete complementarity. For that we utilized the GESS bioinformatics tool to identify possible off-target effects of the two siRNA based only on seed complementarity with the whole transcriptome. A list of potential hits was generated among which FOXN3 deregulation was of interest due to its role

in DNA damage-inducible cell cycle arrest. RT-PCR analysis negated the potential downregulation of this mRNA whose expression was not diminished in either WT or XP-C cells transfected with siLATS1 or siPIK3C3 compared to siAS. c) The downregulation of UVRAG expression post siURAG transfection was validated at the RNA level by PCR as well as protein level by western blot. It should be noted that the level of URAV is already low in XPC cells at the protein level making the decrease of UVAG protein difficult to visualize.

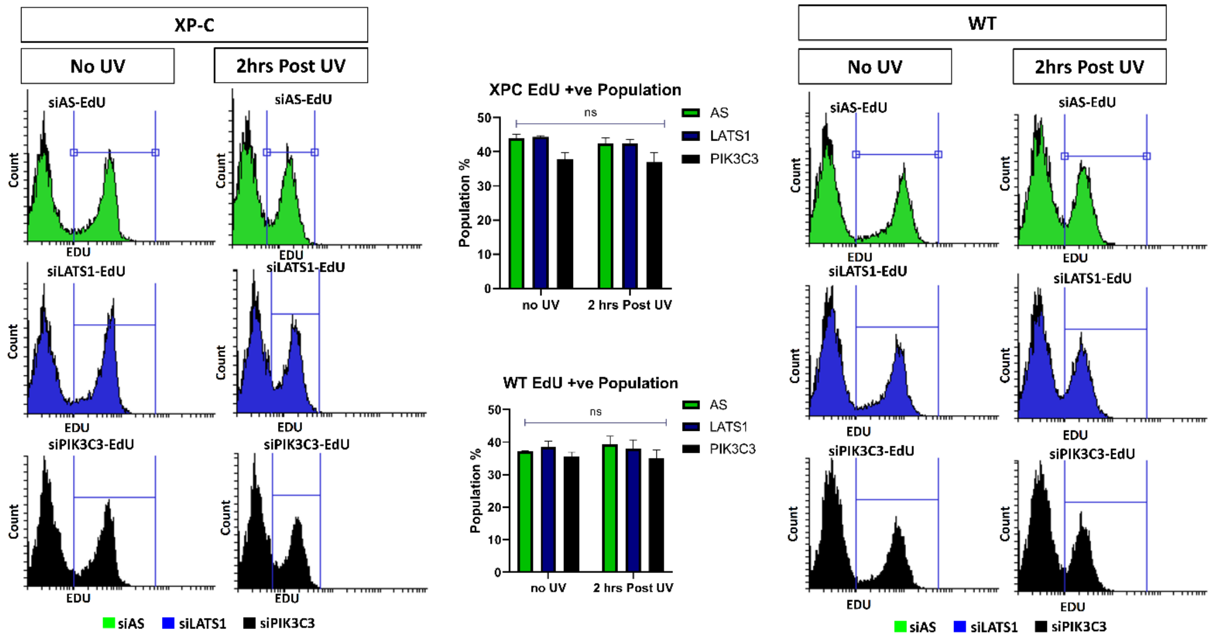

Supplementary Figure2 Same EdU incorporation is evident between siPIK3C3, siLLAST1 and siAS transfected XP-C and WT cell lines.

XP-C or WT cells were transfected with the different siRNAs then irradiated. Prior to the end of the post UVB incubation, the cells were incubated in the presence of EdU. Cells were further on collected, stained and analyzed by flow cytometry. No difference is seen between the EdU positive populations in the different treatment condition at either 2 or 4 hours post UV. Two way anova was used to compare between the two independent variables (time post UV and nature of the siRNA transfected) and the dependent variable of EdU population percentage.

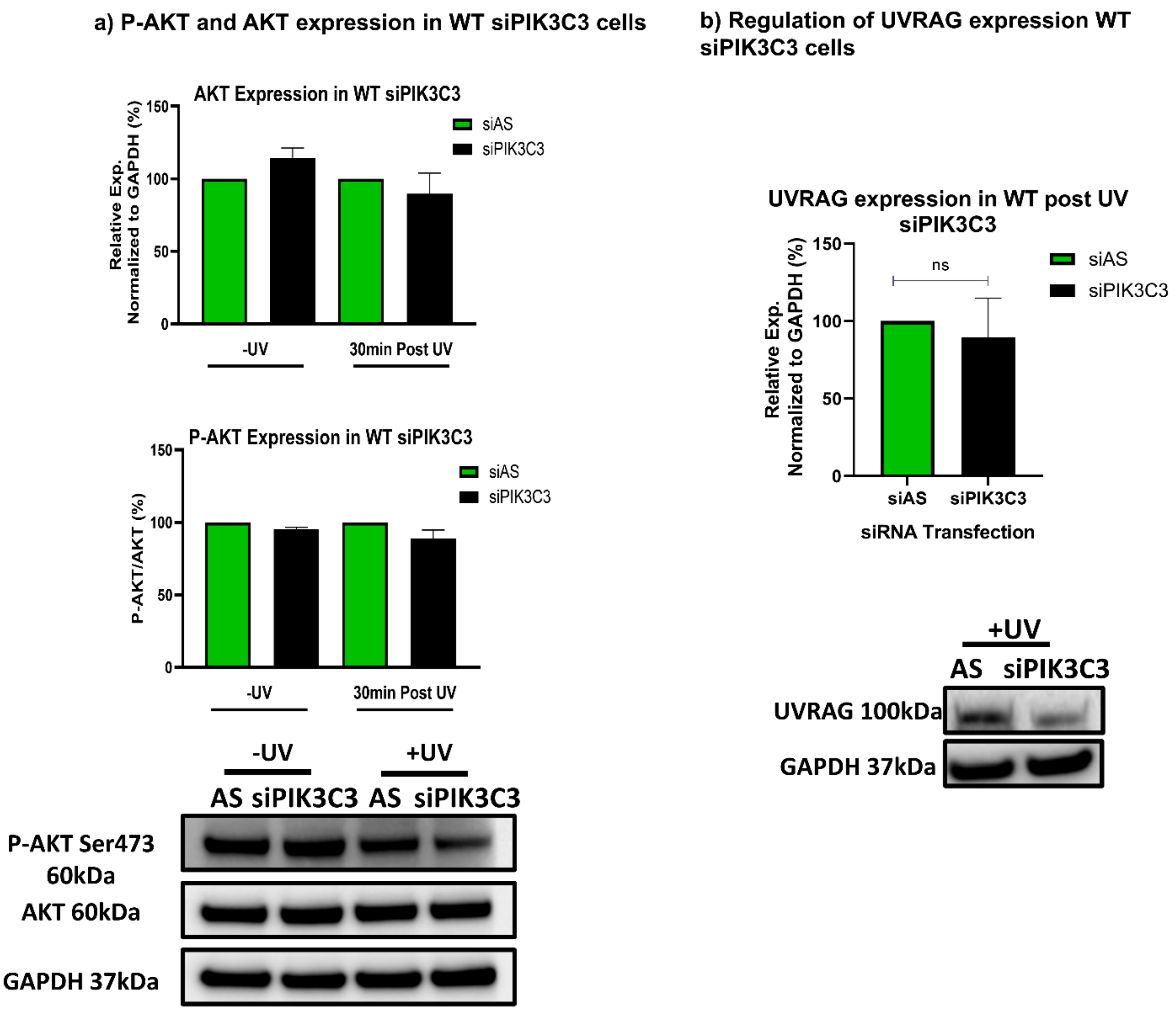

Supplementary Figure3 Downregualtion of PIK3C3 in WT cells manifest differential expression levels of AKT, P-AKT Ser473 and UVRAG compared to XP-C cells.

WT cells were transfected with either siAS or siPIK3C3 then irradiated. Protein extraction was then carried out to follow up the phosphorylation profile of AKT and UVRAG expression .a) P-AKT and AKT expression in WT siPIK3C3 cells. Similarly no difference was also detected for the expression levels and phosphorylation of AKT. b) Regulation of UVRAG expression in WT. UVRAG expression was non-significantly downregulated in WT cells upon siPIK3C3 transfection. Paired t test.

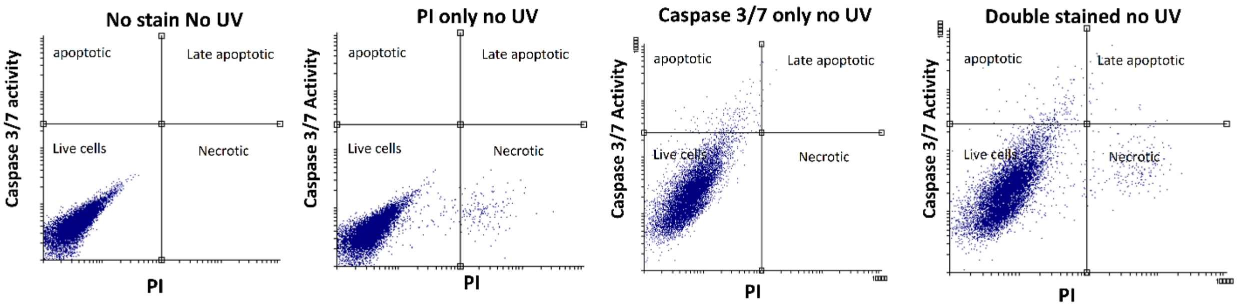

Supplementary Figure 4 Control gating strategy for the quantification of apoptosis with caspase 3/7 activity and necrosis with PI stain

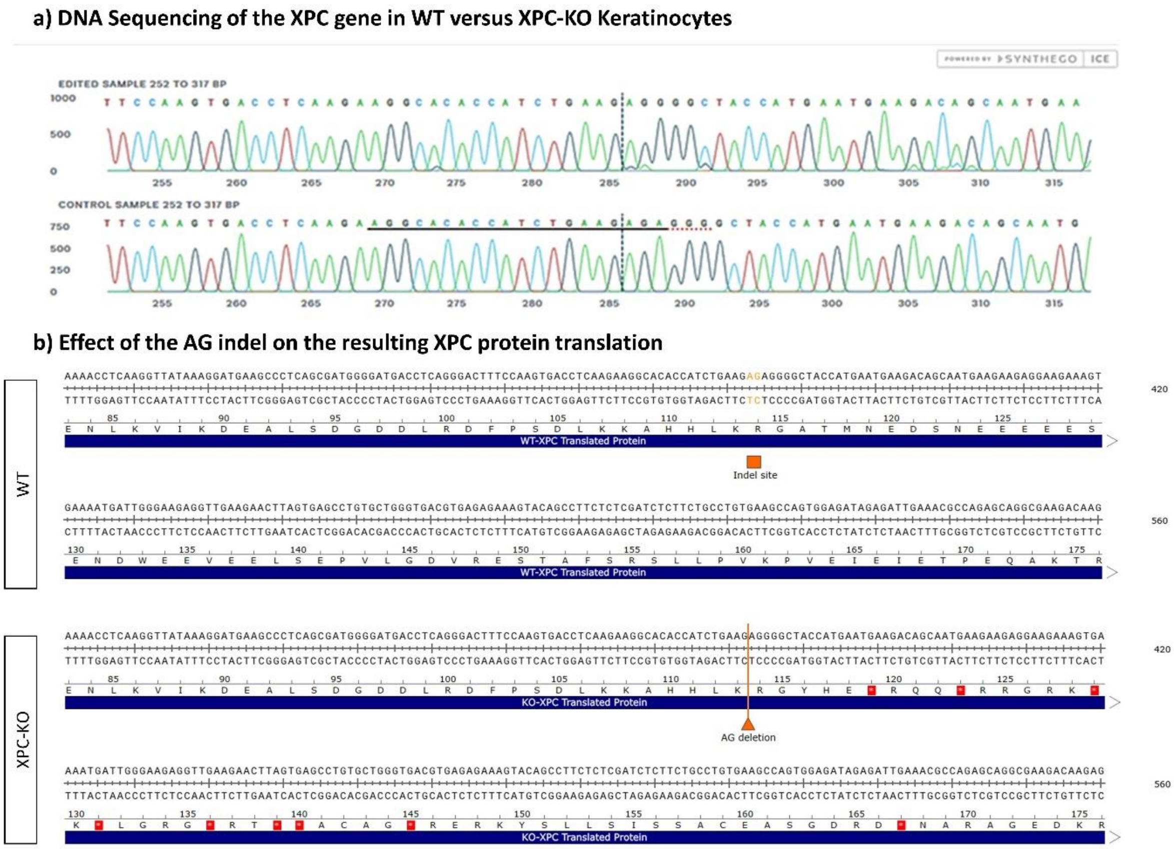

Supplementary Figure 5 Sequencing analysis of N/TERT-2G XPC knockout (KO) compared to wildtype. *The N/TERT-2G XPC knockout (KO) were subjected to Sanger sequencing and compared to the wild-type DNA sequence. a) Comparison of both sequences showed a predominant two-nucleotide (AG) indel mutation, in the exon 3 site b) Insilco translation of the resulting XPC coding sequence revealed the introduction of several stop codons (presented in red) just downstream of the AG indel in the XPC-KO versus WT.*
