# Supplementary material for: Synthetic rescue of Xeroderma Pigmentosum C phenotype via PIK3C3 downregulation
Source: Cell Death Dis. 2024 Nov 19;15(11):847. doi: 10.1038/s41419-024-07186-4 (PMC11577109; doi:10.1038/s41419-024-07186-4)

Figure 1 uncropped Western blots


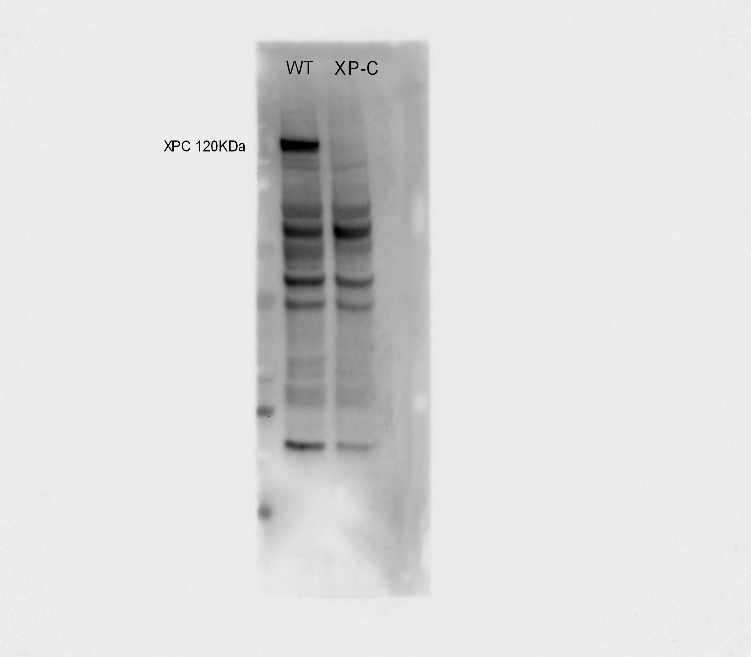

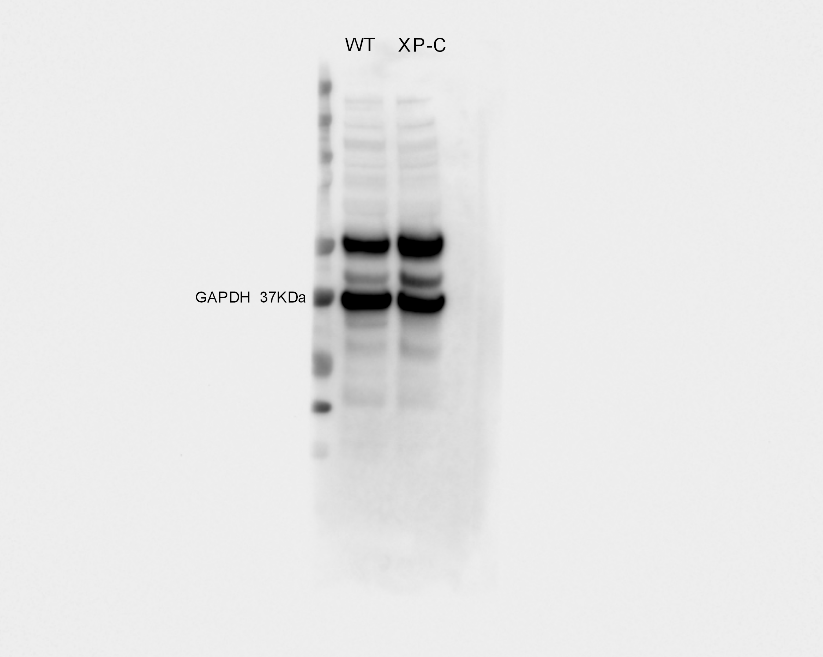


Figure 7 a uncropped western blots


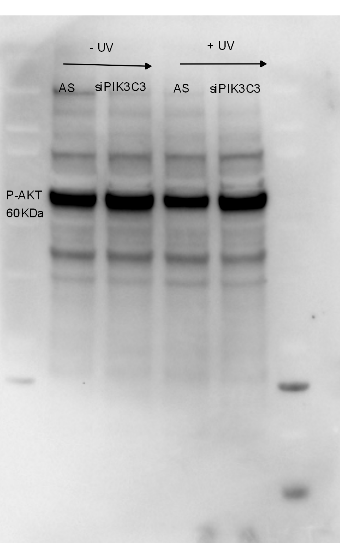

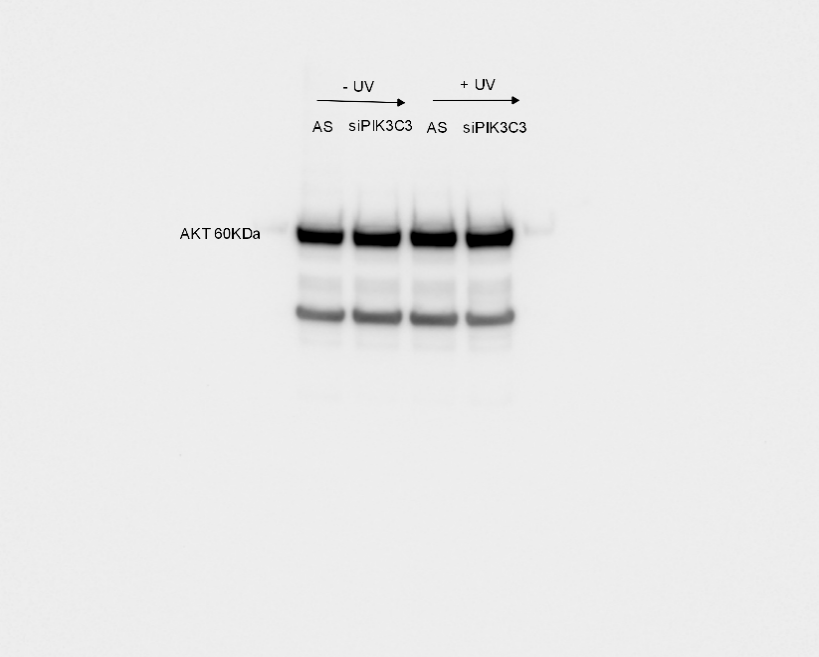

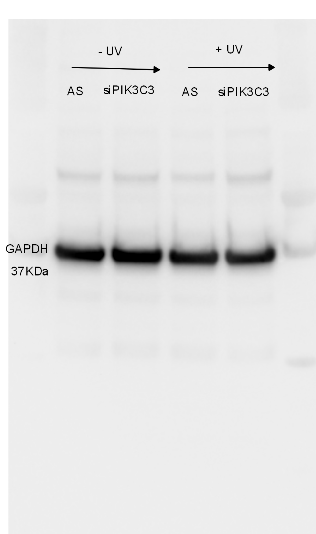


Figure 7c uncropped western blots


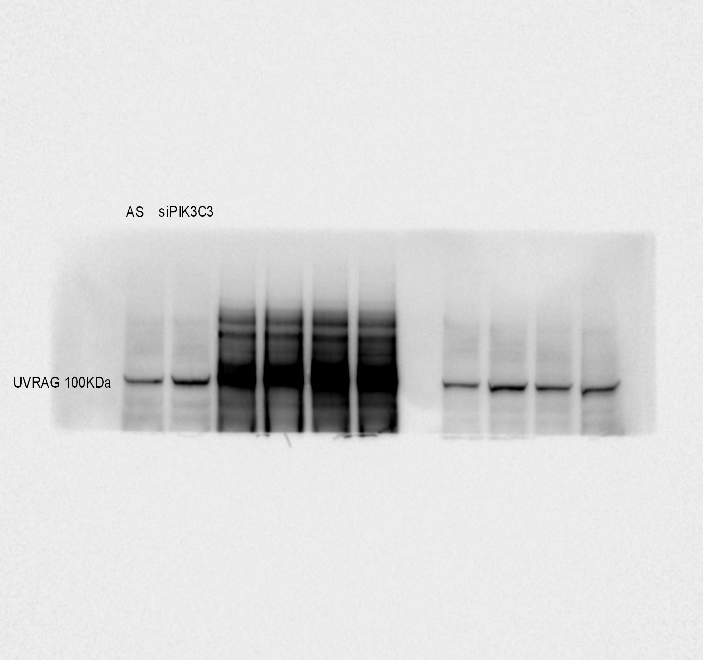

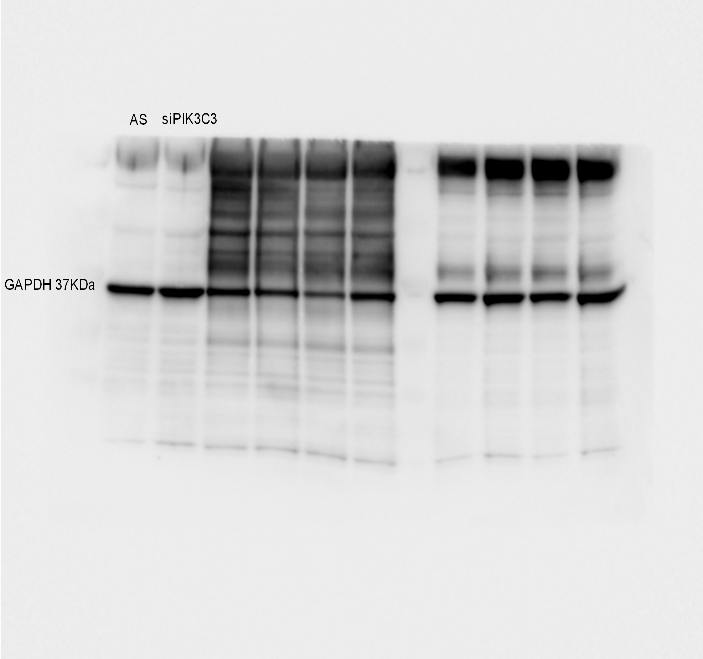

Supplement: Supplementary file 4 — Supplemental uncropped western [file 41419_2024_7186_MOESM4_ESM.docx]
